# Lack of Endogenous Annexin A1 Increases Mast Cell Activation and Exacerbates Experimental Atopic Dermatitis

**DOI:** 10.3390/cells8010051

**Published:** 2019-01-15

**Authors:** Jéssica dos Santos Parisi, Mab Pereira Corrêa, Cristiane Damas Gil

**Affiliations:** 1 Department of Morphology and Genetics, Federal University of São Paulo (UNIFESP), São Paulo 04023-900, Brazil; parisijessica@live.com; 2 Institute of Biosciences, Humanities and Exact Sciences (Ibilce), São Paulo State University (UNESP), São José do Rio Preto 15054-000, Brazil; mabiiic@gmail.com

**Keywords:** inflammation, mast cell, eosinophil, ERK, immunohistochemistry, skin

## Abstract

Annexin A1 (AnxA1) is a protein with potent anti-inflammatory actions and an interesting target that has been poorly explored in skin inflammation. This work evaluated the lack of endogenous AnxA1 in the progression of ovalbumin (OVA)-induced atopic dermatitis (AD)-like skin lesions. OVA/Alum-immunized C57BL/6 male wild-type (WT) and AnxA1 null (AnxA1^-/-^) mice were challenged with drops containing OVA on days 11, 14–18 and 21–24. The AnxA1^-/-^ AD group exhibited skin with intense erythema, erosion and dryness associated with increased skin thickness compared to the AD WT group. The lack of endogenous AnxA1 also increased IgE relative to WT animals, demonstrating exacerbation of the allergic response. Histological analysis revealed intense eosinophilia and mast-cell activation in AD animals, especially in AnxA1^-/-^. Both AD groups increased skin interleukin (IL)-13 levels, while IL-17A was upregulated in AnxA1^-/-^ lymph nodes and mast cells. High levels of phosphorylated ERK were detected in keratinocytes from AD groups. However, phospho-ERK levels were higher in the AnxA1^-/-^ when compared to the respective control groups. Our results suggest AnxA1 as an important therapeutic target for inflammatory skin diseases.

## 1. Introduction

Atopic dermatitis (AD), also known as atopic eczema, is the most common chronic inflammation of skin is characterized by itchy, red, wet and cracked skin [1,2]. AD etiology is multifactorial, and there is evidence that genetic predisposition and family history of atopic disease influences the AD onset. AD pathogenesis is attributed to imbalances in the adaptive immune system, including dysfunction of T helper (Th) lymphocytes and increased production of IgE [1,2,3,4]. Cytokines and chemokines, such as interleukin IL-4, IL-5, IL-13, eotaxins, chemokines CCL17, CCL18 and CCL22, produced by Th2 cells and dendritic cells stimulate infiltration of mast cells and eosinophils into skin [1,2,3]. Th2 and Th17 lymphocytes predominate in patients with AD, but Th1 cells also contribute to its pathogenesis. Th2 and Th17 cells (IL-4/IL-13 and IL-17/IL-22, respectively) produce cytokines that inhibit terminal differentiation of epidermis and contribute to the rupture of the epithelial barrier in AD patients [1,2,4]. In addition, cellular responses triggered during the allergic reaction are controlled by different signaling pathways, such as mitogen-activated protein kinases (MAPKs), which contribute to leukocyte recruitment, mast cell activation, production of proinflammatory cytokines and differentiation of Th2 and Th17 lymphocytes [5,6].

In this scenario, we highlight annexin A1 (AnxA1) as an interesting therapeutic target for skin inflammation. AnxA1 is capable of mimicking the anti-inflammatory action of glucocorticoids by inhibiting the synthesis of eicosanoids and phospholipase A2 (PLA_2_); thus, affecting components of the inflammatory reaction and the release of arachidonic acid [7,8]. In addition, the activity and protective role of the full-length AnxA1 protein as well as its Ac_2-26_ N-terminal peptide, have been explored in several experimental models of acute and chronic inflammation [9,10,11,12].

However, the role of AnxA1 in normal and inflamed skin has been poorly studied. In an oxazolone-induced contact hypersensitivity model, AnxA1 knockout mice (AnxA1^-/-^) presented an exacerbated inflammatory response with increased skin edema and adhesion of CD4+ and CD8+ T cells and neutrophils in the vessels compared to wild-type (WT) animals [13]. The lack of endogenous AnxA1 was associated with increased GATA3 and RORγt transcription factor mRNA expression in the lymph nodes, related to the activation of Th2 and Th17 cells, respectively. In addition, the role of AnxA1 in allergic inflammation has been demonstrated in studies using ovalbumin-induced asthma in mice. One such study showed an association between the pathogenesis of asthma and a decrease in endogenous AnxA1 levels in the lungs, but not of its mRNA, probably related to cleavage of the protein to a 33 kDa isoform [14]. Subsequent investigations demonstrated the anti-allergic activity of an AnxA1-derived peptide (Ac_2-26_) through inhibition of mast cell degranulation and histamine release, and neutrophil/eosinophil accumulation in bronchoalveolar fluid (BAL), despite no change in serum IgE levels and chemotaxis of eosinophils in vitro [15,16]. In contrast, the administration of a permeable AnxA1 protein (Tat- AnxA1) significantly decreased serum IgE, Th2 cytokine levels (IL-4, IL-5 and IL-13) in BAL and inhibited the phosphorylation of ERK, p38 and SAPK/JNK showing fundamental mechanisms of action to attenuate the pathogenesis of the disease [17]. Given the anti-inflammatory properties of AnxA1, we evaluated the effect of a lack of endogenous AnxA1 in a model of atopic dermatitis induced by ovalbumin.

## 2. Materials and Methods

### 2.1. Animals

Male C57BL/6 wild-type and AnxA1-null (AnxA1^-/-^) mice weighing 20–25 g, were kept in cages (*n* = 3–5 animals), in a temperature-controlled environment (22 at 25 °C) and received water and food ad libitum. All animal procedures were approved by the Ethics Committee in Animal Experimentation of the Federal University of São Paulo—UNIFESP (CEUA nº 4910211216) and by the Internal Biosafety Commission (CIBio).

### 2.2. Experimental Protocol of AD Model

WT and AnxA1^-/-^ mice were distributed in three experimental groups: Naïve, Sham and AD. On days 0 and 7, animals were immunized with a subcutaneous injection of 5 µg of ovalbumin (OVA, grade C; Sigma-Aldrich, St Louis, MO, USA) and 10 mg/mL of aluminum hydroxide adjuvant diluted in 200 µL of sterile saline according to previous studies [18]. On day 11, animals were shaved and the hair removed from the entire back. Skin of the mice was challenged with drops containing 250 µg OVA diluted in 50 µL of Johnson’s^®^ baby oil on days 11, 14–18 and 21–24. The Sham group received only sterile saline (days 0 and 7) and oil (days 11, 14–18, 21–24), while the Naïve group animals were only handled. Twenty-four hours after the final OVA challenge, mice were anesthetized with ketamine (100 mg/kg) and xylazine (20 mg/kg) followed by cardiac puncture to obtain blood. Animals were euthanized for skin and cervical lymph node collection.

### 2.3. Analysis of IgE Anti-Ovalbumin and Cytokines

To determine IL-13 and IL-17A cytokine levels by ELISA, skin and cervical lymph nodes of different experimental groups were homogenized in microtubes with a complete cocktail of EDTA-free protease inhibitors (Roche Applied Science, Mannheim, Germany), diluted in lysis buffer (Tris-HCl 50 mM, NaCl 150 mM) and 1% Triton-X, pH 7.4. Finally, samples were centrifuged at 14,000 rpm for 10 min.

IgE anti-OVA levels were measured using a commercially available mouse IgE anti-OVA immunoassay kit (Cayman Chemical Co., Ann Arbor, MI, USA) in accordance with the manufacturer’s instructions. All experiments were conducted in duplicate, and the data expressed as the mean ± SEM protein (ng/mL).

### 2.4. Macroscopy, Skin Thickness, Histopathology and Quantification of Inflammatory Cells

Animals were photographed on the final day of the experimental protocol (day 24) for macroscopic skin analyses. Skins were fixed in 4% paraformaldehyde for 24 h, washed in tap water, dehydrated in a decreasing ethanol series, and embedded in paraffin. Sections of 4 µm were obtained in a Leica RM2155 microtome, deparaffinized and stained with toluidine blue and hematoxylin-eosin for histopathology and quantification of mast cells and eosinophils, respectively.

Eosinophils and mast cells were quantified using a 40× objective on an Axio Scope A1 Zeiss microscope (Carls Zeiss, Jena, Germany). Mast cells were identified according to their metachromatic cytoplasmic granules. Degranulated mast cells were defined as those showing the release of >10% cellular granules. Skin sections analyzed per animal and the area was determined using AxioVision software (Carl Zeiss). Values are expressed as the mean ± SEM cells per mm^2^. Skin thickness (epidermis + dermis) and isolated epidermis were evaluated using photomicrographs taken with a 10× objective. For each animal, three measurements of the epidermis + dermis were taken at random intervals using AxioVision software (Carl Zeiss). Values are shown as mean ± SEM of the thickness (mm) obtained in the different experimental groups.

### 2.5. Immunohistochemistry

Analysis of IL-17A and p-ERK expression was performed on 4 µm sections of paraffin-embedded skin under different experimental conditions in 4% silanized slide preparations. After an antigen retrieval step using citrate buffer (pH 6.0), endogenous peroxide activity was blocked and the sections were incubated overnight at 4 °C with mouse monoclonal anti-p-ERK (Cell Signaling, Danvers, MA, EUA) and rabbit polyclonal anti-IL17A (Peprotech, Rocky Hill, NJ, USA), diluted 1:200 in PBS 1% BSA. After washing, sections were incubated with streptavidin-biotin peroxidase (Histostain SP kit HRP, Invitrogen-Thermo Fisher Scientific, MA, USA) by the development with 3, 3′-diaminobenzidine (DAB, Dako). The slides were counterstained with hematoxylin.

Densitometric analyses for the p-ERK and IL-17A immunostaining were performed on the skin (*n* = 3–5 animals/group), and 50 random points were analyzed in five fields of skin for an average related to the intensity of immunoreactivity [9,11,12]. The values were obtained as arbitrary units (au) between 0 and 255 using AxioVision (Zeiss) software on an AxioSkop 2 microscope (Zeiss, Jena, Germany), with a 40× objective, and the data are expressed as the mean ± SEM of the arbitrary units.

### 2.6. Immunofluorescence

To detect the colocalization of IL-17A and mouse mast cell protease 6 (mMCP6) in mast cells, 4 µm AD skin sections were incubated sequentially with the following reagents at room temperature: (1) distilled water, (2) 4% BSA containing 1% Tween-20 for 1 h, (3) PBS for 5 min, (4) PBS containing 4% BSA and 3% glycine for 1 h, (5) the goat polyclonal antibody anti-mMCP6 (1:300; R&D Systems, Minneapolis, MN, USA) and rabbit polyclonal anti-IL-17A (1:200; Peprotech) for 16–18 h, and (6) three washes (5 min each) in PBS. To detect mMCP6 and IL-17A, FITC-conjugated rabbit anti-goat or PE-conjugated goat anti-rabbit antibodies (1:300; Merck Millipore, Burlington, MA, USA) were added, respectively. After 1 h, the sections were washed extensively in PBS, mounted with Fluoroshield™ containing DAPI (Sigma) and examined using a Nikon Eclipse Ci-S fluorescence microscope.

### 2.7. Statistical Analyses

The data were analyzed using GraphPad Prism 5.0 software. Results were confirmed to follow a normal distribution using the Kolmogorov-Smirnov test of normality with Dallal–Wilkinson–Lillie for corrected *p*-value. Data that passed the normality assumption were analyzed using ANOVA with Bonferroni post hoc test. Data failing the normality assumption were analyzed using the non-parametric Kruskal-Wallis test followed by Dunn’s post-test. Differences were considered statistically significant at a value of *p* < 0.05.

## 3. Results

### 3.1. The Lack of Endogenous AnxA1 Increases Epidermal Thickness and IgE Levels

**Figure 1:**
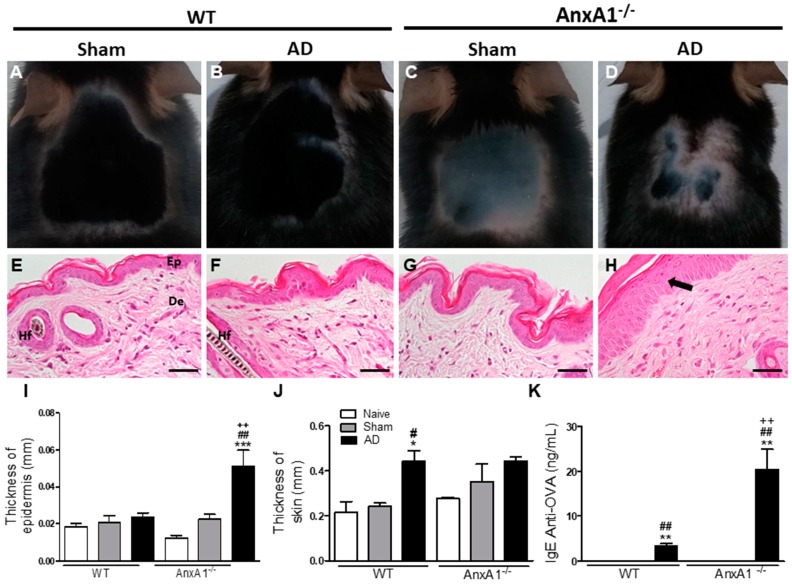
Effect of the absence of endogenous AnxA1 in the AD model. (**A**–**D**): Macroscopic skin analysis. AnxA1^-/-^ mouse from the AD group (**D**) with signs of redness and cutaneous lesions. (**E**–**H**): Microscopic analysis of the skin. Epidermis (Ep) and dermis (De) of normal appearance [E]. Hair follicle (Hf). Epidermal hyperplasia (arrow) is seen in the AnxA1^-/-^ group (**H**). Bars: 40 μm. (**I**,**J**): Thickness of the epidermis and skin. (**K**): IgE anti-ovalbumin levels in plasma. The data represent mean ± S.E.M of skin thickness/epidermis (mm) and IgE levels (ng/mL) in the different experimental groups (*n* = 3–5 animals/group). * *p* < 0.05, ** *p* < 0.01, *** *p* < 0.001 vs. Naïve of the respective genotype; # *p* < 0.05, ## *p* < 0.01 vs. Sham of the respective genotype; ++ *p* < 0.01 vs. AD WT (ANOVA, Bonferroni post test).

We analyzed the allergic inflammatory response of mouse skins on the final day of the experimental protocol (day 24) by macroscopic and histological methods. The AnxA1^-/-^ AD group presented skin with signs of redness and more intense lesions than the WT AD group. Control groups (Naïve and Sham) of both genotypes showed homogenous growth with no lesions (Figure 1A–D). There was notable epidermal hyperplasia in AnxA1^-/-^ animals from the AD group in relation to their respective controls and the WT AD group, corroborating the macroscopic analyses (Figure 1E–I). In contrast, WT animals from the AD group presented greater skin thickness than their respective controls (Figure 1J). WT and AnxA1^-/-^ animals from the AD group showed increased serum anti-OVA IgE levels in relation to the respective Naïve and Sham controls (Figure 1K). In addition, the lack of AnxA1 endogenous caused a significant increase of IgE in relation to the WT animals, suggesting an exacerbation of the allergic response (Figure 1K).

### 3.2. Effect of the Lack of Endogenous AnxA1 on the Inflammatory Response of the Skin

**Figure 2:**
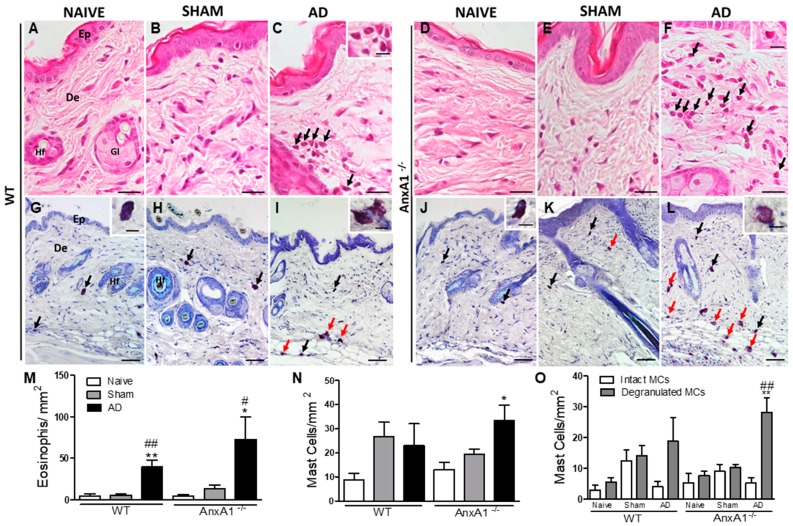
Histopathology of the skin. (**A**,**B**,**D**,**E**,**G**,**H**,**J**,**K**): WT and AnxA1^-/-^ control skins (Naïve and Sham). Atopic dermatitis (AD) characterized by intense influx of eosinophils (arrows) (**C**,**F**) and intact and degranulated mast cells (black and red arrows, respectively; I, L) into the dermis. Insets: detail of eosinophils (**C**,**F**), intact (**G**,**J**) and degranulated mast cells (**I**,**L**). Epidermis (Ep). Dermis (De). Hair follicle (Hf). Sebaceous gland (Gl). Stain: Hematoxylin-eosin (**A**–**F**) and toluidine blue (**G**–**L**). Bars: 20 μm (**A**–**F**), 50 μm (**G**–**L**), insets: 10 μm. (**M**): Quantification of eosinophils in the skin. (**N**): Quantification of the total number of mast cells in the skin. Data represent mean ± SEM of the number of cells per mm^2^ of the experimental groups (*n* = 3–5 animals/group). * *p* < 0.05; ** *p* < 0.01 vs. Naïve of the respective genotype; # *p* < 0.05; ## *p* < 0.01 vs. Sham of respective genotype (ANOVA, Bonferroni post test). (**O**): Quantification of intact and degranulated mast cells. ** *p* < 0.01 vs. intact mast cells of the AnxA1^-/-^ AD group, ## *p* < 0.01 vs. degranulated mast cells from the AnxA1^-/-^ Naïve and Sham groups (ANOVA, Bonferroni post test).

Histopathological analysis of the skin of control, Naïve and Sham animals, revealed normal-looking skin with no morphological changes to the epidermis or dermis (Figure 2A,B,D,E). However, the animals sensitized and challenged with OVA presented an inflammatory response characterized by the presence of inflammatory cells in the dermis, mainly eosinophils and degranulated mast cells (Figure 2A–L).

Quantification of these inflammatory cells confirmed the histological analysis. WT and AnxA1^-/-^ animals from the AD groups showed a significant increase of eosinophils in the skin (Figure 2M) relative to their respective controls (Naïve and Sham). In addition, a significant increase in the total number of mast cells was detected only in the AnxA1^-/-^ AD group compared to the respective control groups (Figure 2N). Morphological analysis of mast cell populations differentiated into intact and degranulated cells revealed a significant increase in the population of activated mast cells in the AnxA1^-/-^ group (Figure 2O). The number of degranulated mast cells in the AnxA1^-/-^ animals of the AD group was significantly higher than the respective Naïve and Sham groups of the same genotype. Furthermore, there was a significant difference between intact and degranulated cell numbers in the AnxA1^-/-^ group (Figure 2O).

We also observed a marked increase in IL-13 levels in the skin homogenates of AD animals of both genotypes compared to their respective control groups (AD WT: 1.17 ± 0.09 ng/mL, AD AnxA1^-/-^: 1.24 ± 0.23 ng/mL, *p* < 0.001 vs. Naïve and Sham of both genotypes). No alterations in IL-13 levels were found in lymph nodes of any group (data not shown). However, the lack of AnxA1 produced a significant increase in IL-17A levels in the lymph nodes in relation to the control animals of the same genotype (AD: 1.88 ± 0.31 ng/mL, *p* < 0.001 vs. Naïve and Sham). No alterations in IL-17A levels were evident in the skin homogenates of AnxA1^-/-^ groups, while WT mice showed no detectable levels of this cytokine in the lymph nodes and skin homogenates (data not shown).

### 3.3. AD Positively Modulates IL-17A and p-ERK Levels in the Skin

**Figure 3:**
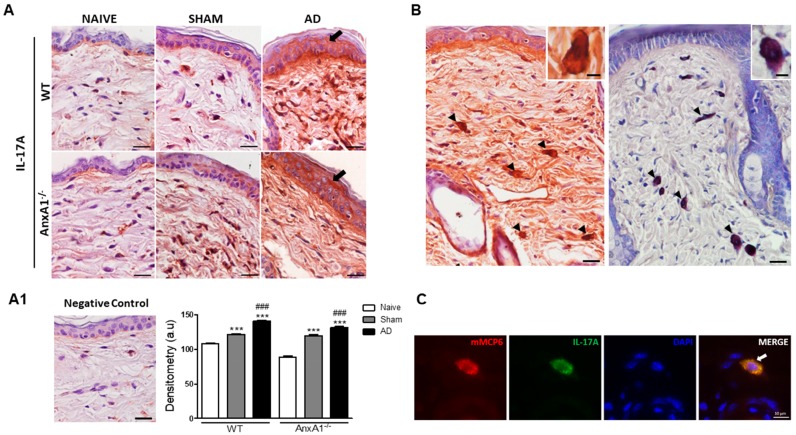
Expression of IL-17A in the skin. (**A**): WT and AnxA1^-/-^ AD with intense IL-17A immunoreactivity in the epidermis (arrows) compared to the Naïve and Sham control groups of the respective genotypes. Counterstain: Hematoxylin. Bars: 20 μm. (**A1**): Absence of immunoreactivity in the negative control of the reaction. IL-17A densitometry in the epidermis. Data represent mean ± SEM of IL-17A expression in arbitrary units (a.u.) (*n* = 3–5 animals/group). *** *p* < 0.001 vs. Naïve of the respective genotype; ^###^ *p* < 0.001 vs. Sham of the respective genotype (Kruskall–Wallis, Dunn post test). (**B**): IL-17A-positive cells (arrowheads) in the dermis showed similar aspect of metachromatic mast cells (arrows) in the dermis. Insets: detail of IL-17A-positive cells and mast cells. Counterstain: Hematoxylin. Stain: Toluidine blue. Bars: 20 μm; 5 μm (insets). (**C**): Double-staining for mMCP6 (red) and IL-17A (green) in AnxA1^-/-^ skin. mMCP6 and IL-17A are co-localized in the cytoplasmic granules of mast cells (arrow). DAPI was used as nuclear counterstain. Bar: 10 μm.

Due to the AnxA1^-/-^ animals having higher IL-17A levels than WT organ homogenates, we evaluated the expression of this cytokine in skin by immunohistochemistry. Intense immunoreactivity was detected for IL-17A in epidermis of WT and AnxA1^-/-^ animals from the AD group relative to their respective Naïve and Sham controls (Figure 3A). We also observed greater IL-17A immunoreactivity in Sham animals of both genotypes compared to both Naïve groups. Densitometry confirmed histological observations showing a marked IL-17A increase in the epidermis of WT and AnxA1^-/-^ AD group animals compared to controls (Figure 3A1). No immunoreactivity was detected in the negative control (Figure 3A1). In the dermis of all experimental groups, we also observed the presence of positive IL-17A cells very similar to the mast cells, as demonstrated by toluidine blue stain (Figure 3B). Immunofluorescence analysis confirmed this finding, showing co-localization of mMCP6 and IL-17A in the cytoplasm of mast cells (Figure 3C).

**Figure 4:**
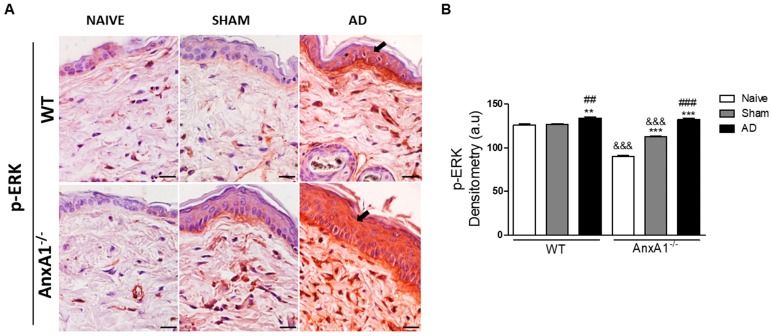
p-ERK levels in the skin. (**A**): WT and AnxA1^-/-^ AD with intense p-ERK immunoreactivity in the epidermis (arrows) compared to the Naïve and Sham control groups of the respective genotypes. Bars: 20 μm. (**B**): Densitometric analysis of p-ERK in the epidermis. Data represent mean ± SEM of p-ERK in arbitrary units (a.u.) (*n* = 3–5 animals/group). ** *p* < 0.01, *** *p* < 0.001 vs. Naïve of respective genotype; ^##^ *p* < 0.01, ^###^ *p* < 0.001 vs. Sham of the respective genotype; ^&&&^ *p* < 0.001 vs. corresponding WT groups (Kruskall–Wallis, Dunn post test).

Finally, p-ERK levels were detected in the epidermis and in the cells present in the dermis (Figure 4). WT and AnxA1^-/-^ AD groups showed intense immunoreactivity for p-ERK in the epidermis compared to their respective controls (Figure 4A). Densitometric analysis confirmed a marked increase of p-ERK levels in the epidermis of both AD group genotypes. This increase was exacerbated in the AnxA1^-/-^ mice when compared to their respective controls (Figure 4B). Additionally, the lack of AnxA1 was associated with reduced p-ERK levels in the epidermis of controls (Naïve and Sham) compared to WT (Figure 4B).

## 4. Discussion

In the present study, we evaluated the effect of the absence of endogenous AnxA1 on OVA-induced AD in mice. Using macroscopic, histological, biochemical and immunohistochemical analyses, we show that AnxA1 is an important anti-inflammatory mediator in the allergic AD skin response.

Initially, we verified that the AnxA1^-/-^ AD group presented skin with signs of redness and more intense lesions, as well as a significant increase in epidermal thickness compared to the WT AD group. In addition, the absence of endogenous AnxA1 caused a significant increase of plasma anti-OVA IgE levels compared to WT animals, suggesting an exacerbation of the allergic response. Histological analysis revealed intense eosinophilia and activation of mast cells in AD animals of both genotypes. The macroscopic and histological characteristics observed in our study are consistent with other experimental models of AD induced by OVA [18,19,20], 2,4-dinitrochlorobenzene [21], house-dust mite [22] and oxazolone [23], which mimic the type I and IV inflammatory hypersensitivity responses.

The absence of endogenous AnxA1 in the AD group was associated with a significant increase in mast cell numbers in the dermis when compared to the respective control groups and corroborated by high levels of plasma anti-OVA IgE. Furthermore, we observed a higher proportion of degranulated mast cells compared to Naïve and Sham controls. Activated or not by anti-IgE antibodies, mast cells from AnxA1^-/-^ mice are more susceptible to degranulation and release of histamine and prostaglandins than WT mouse cells in response to different stimuli (zymosan A, compound 48/80 and nedocromil) in vitro [24,25]. In addition, pre-treatment of mice with AnxA1 in OVA-induced pleuritis has been shown to significantly increase the number of intact mast cells in the pleural fluid and, in vitro, decrease histamine release [15]. Similarly, treatment with Ac_2-26_ reduced the proportion of degranulated mast cells compared to the non-treated mice with OVA-induce allergic conjunctivitis [11,12]. The Ac_2-26_ effect on mast cell degranulation was reversed after combined treatment with formyl peptide receptor (FPR) antagonist Boc2 [12]. Moreover, AnxA1 was shown to co-localize with FPR2 in the plasma membrane of mast cells [12] and administration of an FPR2 antagonist, WRW4, inhibited mast cell degranulation [26]. These findings indicate that the AnxA1-Fpr system is functional in mast cells during the allergic response.

We also observed an increase in IL-13 levels in the skin of animals from the AD group, with no difference seen between the various genotypes. However, the absence of endogenous AnxA1 significantly increased lymph node IL-17A levels. In a model of contact hypersensitivity, AnxA1^-/-^ animals showed increased proliferation of CD4+ T cells and Th1 and Th17 cytokines [13]. Another study showed that IL-17A receptor gene knockout animals developed spontaneous skin lesions. Thus, evidencing the importance of the IL-17A/IL-17AR axis in inflammatory skin diseases such as AD [27]. In addition, mice with imiquimod-induced psoriatic dermatitis exhibit increased IL-17 mRNA levels and mast cell numbers in the skin [28]. Elevated plasma IL-17 levels have been detected in patients with AD compared to individuals without the disease, reinforcing the role of this cytokine as an important AD biomarker [29,30].

In addition, we found strong immunoreactivity for IL-17A in the epidermis of WT and AnxA1^-/-^ AD animals relative to their respective controls. We also noticed the presence of IL-17A-positive mast cells, implicating these cells as a potential source of this cytokine in AD. In addition to inhibiting the terminal differentiation of the epidermis, IL-17 and IL-22 induce epidermal hyperplasia [31]. Thus, the lack of endogenous AnxA1, high IL-17A levels in the lymph nodes and epidermis and increased number of 1L-17A^+^ mast cells may contribute to the exacerbation of AD and consequent epidermal thickening.

Finally, phospho-ERK levels were exacerbated in the AnxA1^-/-^ animals, when compared to the respective control groups. ERK plays a crucial role in IL-4 production during differentiation of TCR-induced naïve CD4+ T cells to TCR, and the decrease in its activity leads to a decrease in IL-4 production [32]. Furthermore, in an OVA-induced asthma model, the use of an ERK-specific inhibitor caused a decrease in the release of Th2 cytokines, serum immunoglobulins and eotaxin levels in BAL [33]. Thus, in our experimental model, endogenous AnxA1 may represent an important regulator for the upstream ERK activation cascade and consequent cytokine release during AD.

## 5. Conclusions

In conclusion, AnxA1 plays an important role in the anti-allergic response in OVA-induced AD by regulating the production of IgE and IL-17A and recruitment and activation of inflammatory cells, especially mast cells, to the lesion site. Thus, AnxA1 represents a relevant therapeutic target for the AD-like skin lesions in mouse model.

## References

[1] Leung D.Y., Guttman-Yassky E. (2014). Deciphering the complexities of atopic dermatitis: Shifting paradigms in treatment approaches. J. Allergy Clin. Immunol..

[2] Schlapbach C., Simon D. (2014). Update on skin allergy. Allergy.

[3] Dhingra N., Gulati N., Guttman-Yassky E. (2013). Mechanisms of contact sensitization offer insights into the role of barrier defects vs. intrinsic immune abnormalities as drivers of atopic dermatitis. J. Investig. Dermatol..

[4] Garg N., Silverberg J.I. (2015). Epidemiology of childhood atopic dermatitis. Clin. Dermatol..

[5] Pelaia G., Cuda G., Vatrella A., Gallelli L., Caraglia M., Marra M., Abbruzzese A., Caputi M., Maselli R., Costanzo F.S. (2005). Mitogen-activated protein kinases and asthma. J. Cell Physiol..

[6] Acciani T.H., Suzuki T., Trapnell B.C., Le Cras T.D. (2016). Epidermal growth factor receptor signalling regulates granulocyte-macrophage colony-stimulating factor production by airway epithelial cells and established allergic airway disease. Clin. Exp. Allergy.

[7] Flower R.J. (1988). Eleventh Gaddum memorial lecture. Lipocortin and the mechanism of action of the glucocorticoids. Br. J. Pharmacol..

[8] Perretti M., Gavins F.N. (2003). Annexin 1: An endogenous anti-inflammatory protein. News Physiol. Sci..

[9] Girol A.P., Mimura K.K.O., Drewes C.C., Boonheis S.M., Solito E., Farsky S.H.P., Gil C.D., Oliani S.M. (2013). Anti-inflammatory mechanisms of the annexin A1 protein and its mimetic peptide Ac2-26 in models of ocular inflammation in vivo and in vitro. J. Immunol..

[10] Fredman G., Kamaly N., Spolitu S., Milton J., Ghorpade D., Chiasson R., Kuriakose G., Perretti M., Farokzhad O., Tabas I. (2015). Targeted nanoparticles containing the proresolving peptide Ac2-26 protect against advanced atherosclerosis in hypercholesterolemic mice. Sci. Transl. Med..

[11] Gimenes A.D., Andrade T.R., Mello C.B., Ramos L., Gil C.D., Oliani S.M. (2015). Beneficial effect of annexin A1 in a model of experimental allergic conjunctivitis. Exp. Eye Res..

[12] Marmorato M.P., Gimenes A.D., Andrade F.E.C., Oliani S.M., Gil C.D. (2018). Involvement of the annexin A1-Fpr anti-inflammatory system in the ocular allergy. Eur. J. Pharmacol..

[13] Yang Y.H., Song W., Deane J.A., Kao W., Ooi J.D., Ngo D., Kitching A.R., Morand E.F., Hickey M.J. (2013). Deficiency of annexin A1 in CD4+ T cells exacerbates T cell-dependent inflammation. J. Immunol..

[14] Chung Y.W., Oh H.Y., Kim J.Y., Kim J.H., Kim I.Y. (2004). Allergen-induced proteolytic cleavage of annexin-1 and activation of cytosolic phospholipase A2 in the lungs of a mouse model of asthma. Proteomics.

[15] Bandeira-Melo C., Bonavita A.G., Diaz B.L., Silva P.M.R., Carvalho V.F., Jose P.J., Flower R.J., Perretti M., Martins M. (2005). A novel effect for annexin 1-derived peptide ac2-26: Reduction of allergic inflammation in the rat. J. Pharmacol. Exp. Ther..

[16] Wang L.M., Li W.H., Xu Y.C., Wei Q., Zhao H., Jiang X.F. (2011). Annexin 1-derived peptide Ac2-26 inhibits eosinophil recruitment in vivo via decreasing prostaglandin D_2_. Int. Arch. Allergy Immunol..

[17] Lee S.H., Kim D.W., Kim H.R., Woo S.J., Kim S.M., Jo H.S., Jeon S.G., Cho S.W., Park J.H., Won M.H. (2012). Anti-inflammatory effects of Tat-Annexin protein on ovalbumin-induced airway inflammation in a mouse model of asthma. Biochem. Biophys. Res. Commun..

[18] Corrêa M.P., Andrade F.E.C., Gimenes A.D., Gil C.D. (2017). Anti-inflammatory effect of galectin-1 in a murine model of atopic dermatitis. J. Mol. Med..

[19] Kim H.J., Kim Y.J., Kang M.J., Seo J.H., Kim H.Y., Jeong S.K., Lee S.H., Kim J.M., Hong S.J. (2012). A novel mouse model of atopic dermatitis with epicutaneous allergen sensitization and the effect of Lactobacillus rhamnosus. Exp. Dermatol..

[20] Baek J.O., Roh J.Y., Jung Y. (2017). Oral tolerance inhibits atopic dermatitis-like type 2 inflammation in mice by modulating immune microenvironments. Allergy.

[21] Kim H., Kim J.R., Kang H., Choi J., Yang H., Lee P., Kim J., Lee K.W. (2014). 7,8,4′-Trihydroxyisoflavone attenuates DNCB-induced atopic dermatitis-like symptoms in NC/Nga mice. PLoS ONE.

[22] Matsuoka H., Maki N., Yoshida S., Arai M., Wang J., Oikawa Y., Ikeda T., Hirota N., Nakagawa H., Ishii A. (2003). A mouse model of the atopic eczema/dermatitis syndrome by repeated application of a crude extract of house-dust mite Dermatophagoides farinae. Allergy.

[23] Heo W.I., Lee K.E., Hong J.Y., Kim M.N., Oh M.S., Kim Y.S., Kim K.W., Kim K.E., Sohn M.H. (2015). The role of interleukin-17 in mouse models of atopic dermatitis and contact dermatitis. Clin. Exp. Dermatol..

[24] Yazid S., Ayoub S.S., Solito E., McArthur S., Vo P., Dufton N., Flower R.J. (2011). Anti-allergic drugs and the Annexin-A1 system. Pharmacol. Rep..

[25] Yazid S., Sinniah A., Solito E., Calder V., Flower R.J. (2013). Anti-allergic cromones inhibit histamine and eicosanoid release from activated human and murine mast cells by releasing Annexin A1. PLoS ONE.

[26] Pundir P., Catalli A., Leggiadro C., Douglas S.E., Kulka M. (2014). Pleurocidin, a novel antimicrobial peptide, induces human mast cell activation through the FPRL1 receptor. Mucosal Immunol..

[27] Floudas A., Saunders S.P., Moran T., Schwartz C., Hams E., Fitzgerald D.C., Johnston J.A., Ogg G.S., McKenzie A.N., Walsh P.T. (2017). IL-17 Receptor A Maintains and Protects the Skin Barrier To Prevent Allergic Skin Inflammation. J. Immunol..

[28] Cho K.A., Park M., Kim Y.H., Woo S.Y. (2017). Th17 cell-mediated immune responses promote mast cell proliferation by triggering stem cell factor in keratinocytes. Biochem. Biophys. Res. Commun..

[29] Ma L., Xue H.B., Guan X.H., Shu C.M., Wang F., Zhang J.H., An R.Z. (2014). The Imbalance of Th17 cells and CD4(+) CD25(high) Foxp3(+) Treg cells in patients with atopic dermatitis. J. Eur. Acad. Dermatol. Venereol..

[30] Leonardi S., Cuppari C., Manti S., Filippelli M., Parisi G.F., Borgia F., Briuglia S., Cannavo P., Salpietro A., Arrigo T. (2015). Serum interleukin 17, interleukin 23, and interleukin 10 values in children with atopic eczema/dermatitis syndrome (AEDS): Association with clinical severity and phenotype. Allergy Asthma Proc..

[31] Vakharia P.P., Silverberg J.I. (2017). Monoclonal Antibodies for Atopic Dermatitis: Progress and Potential. BioDrugs.

[32] Jorritsma P.J., Brogdon J.L., Bottomly K. (2003). Role of TCR-induced extracellular signal-regulated kinase activation in the regulation of early IL-4 expression in naive CD4+ T cells. J. Immunol..

[33] Duan W., Chan J.H., Wong C.H., Leung B.P., Wong W.S. (2004). Anti-inflammatory effects of mitogen-activated protein kinase kinase inhibitor U0126 in an asthma mouse model. J. Immunol..

